# Sintering, Microstructure, and Dielectric Properties of Copper Borates for High Frequency LTCC Applications

**DOI:** 10.3390/ma14144017

**Published:** 2021-07-18

**Authors:** Dorota Szwagierczak, Beata Synkiewicz-Musialska, Jan Kulawik, Norbert Pałka

**Affiliations:** 1 Łukasiewicz Research Network–Institute of Microelectronics and Photonics, Kraków Division, Zabłocie 39, 30-701 Kraków, Poland; beata.synkiewicz.musialska@imif.lukasiewicz.gov.pl (B.S.-M.); jan.kulawik@imif.lukasiewicz.gov.pl (J.K.); 2 Institute of Optoelectronics, Military University of Technology, ul. gen. S. Kaliskiego 2, 00-908 Warszawa, Poland; norbert.palka@wat.edu.pl

**Keywords:** microelectronics, packaging, copper borates, ceramic substrates, low dielectric permittivity, dielectric properties, THz spectroscopy, LTCC applications

## Abstract

New ceramic materials based on two copper borates, CuB_2_O_4_ and Cu_3_B_2_O_6_, were prepared via solid state synthesis and sintering, and characterized as promising candidates for low dielectric permittivity substrates for very high frequency circuits. The sintering behavior, composition, microstructure, and dielectric properties of the ceramics were investigated using a heating microscope, X-ray diffractometry, scanning electron microscopy, energy dispersive spectroscopy, and terahertz time domain spectroscopy. The studies revealed a low dielectric permittivity of 5.1–6.7 and low dielectric loss in the frequency range 0.14–0.7 THz. The copper borate-based materials, owing to a low sintering temperature of 900–960 °C, are suitable for LTCC (*low temperature cofired ceramics*) applications.

## 1. Introduction

Modern high frequency communication systems create demand for new substrate materials with specific dielectric characteristics comprising a low dielectric permittivity, a low dielectric loss, and a low temperature coefficient of dielectric permittivity. Such dielectric properties of a substrate for microwave and mm-wave circuits improve the signal speed and quality, selectivity, and temperature stability of the operating frequency [1,2,3,4,5]. 

Silicates, such as cordierite, forsterite, diopside, mullite, and willemite, are well-known conventional materials for low dielectric permittivity substrates for microwave circuits [6,7,8,9,10,11,12]. Recently, with the tendency to decrease the sintering temperature, new materials have attracted more attention. Besides molybdates, tungstates, and phosphates, some borates are good candidates for new materials with a low sintering temperature [13,14,15,16,17,18,19,20,21,22,23,24].

The copper metaborate CuB_2_O_4_ crystallizes in a tetragonal structure with I-42d space group [25,26,27]. Its structure is composed of a BO_4_ tetrahedra sharing four common oxygen ions. Cu^2+^ ions are situated between them in two different crystallographic positions corresponding to a planar square or an elongated octahedral coordination [25,26,27].

Cu_3_B_2_O_6_ has a more complex structure and a lower symmetry [28] as compared with CuB_2_O_4_. The best known is Cu_3_B_2_O_6_ with a triclinic structure, although the crystallization of this compound in the monoclinic and orthorhombic structures was also reported. In Cu_3_B_2_O_6_, Cu^2+^ ions occupy 16 nonequivalent crystallographic positions, which can be divided into the following three types—predominant square planar positions (CuO_4_) with the coordination number four, distorted square pyramids (CuO_5_) with the coordination number five, and distorted octahedral positions (CuO_6_) with the coordination number six. For this compound, the calculated average effective coordination number is close to four for the triclinic structure. In Cu_3_B_2_O_6_, boron also shows different coordination numbers—(BO_3_) and (B_2_O_5_) groups occur with shorter B–O bonds than for tetrahedral (BO_4_) groups in CuB_2_O_4_ [28].

Besides broad studies of the magnetic properties of CuB_2_O_4_ and Cu_3_B_2_O_6_ crystals, potential magneto-optical, piezoelectric, multiferroic, and photocatalytic applications of CuB_2_O_4_ were also reported [25,26,27,29,30,31,32,33,34,35,36,37]. Furthermore, Cu_3_B_2_O_6_ was applied for active electrodes of lithium-ion batteries [38,39]. However, the characterization of dielectric properties at THz frequencies for ceramic substrates made of copper borates has remained an unexplored area thus far.

This work reports on sintering behavior, microstructure, and dielectric properties in the THz range of new ceramics based on two pure copper borates, CuB_2_O_4_ and Cu_3_B_2_O_6_, and CuB_2_O_4_–Cu_3_B_2_O_6_ mixtures. These ceramics offer a low dielectric permittivity and a low dielectric loss at very high frequencies, and a relatively low sintering temperature adequate for LTCC (*low temperature cofired ceramics*) technology.

## 2. Materials and Methods

Two copper borates, CuB_2_O_4_ and Cu_3_B_2_O_6_, were synthesized using the conventional solid state reaction method. The high purity starting materials, H_3_BO_3_ and CuO (Sigma Aldrich, St. Louis, MO, USA), were mixed in stoichiometric proportions, ball milled (Pulverisette 5, Fritsch, Germany) for 8 h in isopropyl alcohol, and dried. Then, the powders were pressed into pellets and calcined in a two-step process—at 200–400 °C for 2 h to decompose boric acid, and at 700 °C for 5 h to carry out solid state syntheses.

The resulting materials were ball milled for 8 h to obtain fine CuB_2_O_4_ and Cu_3_B_2_O_6_ powders. In addition, three CuB_2_O_4_–Cu_3_B_2_O_6_ mixtures containing 35, 50, and 70 wt.% Cu_3_B_2_O_6_ were prepared by ball milling for 8 h. For the last two compositions, 5 wt.% CuBi_2_O_4_ was added as a sintering aid. Finally, the powders were granulated with polyvinyl alcohol, pressed into pellets, and sintered in the temperature range 900–960 °C.

The phase compositions of the materials were investigated using the X-ray diffraction method (Empyrean, PANalytical, Almelo, The Netherlands) using Cu K_α1_ radiation within a 2Ɵ range of 10 to 90°. Optimal sintering conditions and melting points of the samples were established based on observations in a heating microscope (Leitz, Germany) in the temperature range 20–1040 °C. Scanning electron microscopy and X-ray energy dispersive spectroscopy (FEI Nova Nano SEM 200 with EDAX Genesis EDS system, Hillsboro, OR, USA) were used to characterize the microstructure and elemental composition of the ceramics.

Dielectric properties at room temperature in the frequency range 0.12–2.5 THz were studied using time domain spectroscopy (TDS) (TPS Spectra 3000, Teraview, Cambridge, UK) according to the procedure reported previously [12]. The measurements were performed in purged air to avoid interference related to the presence of water vapor.

## 3. Results and Discussion

### 3.1. Phase Composition

**Figure 1:**
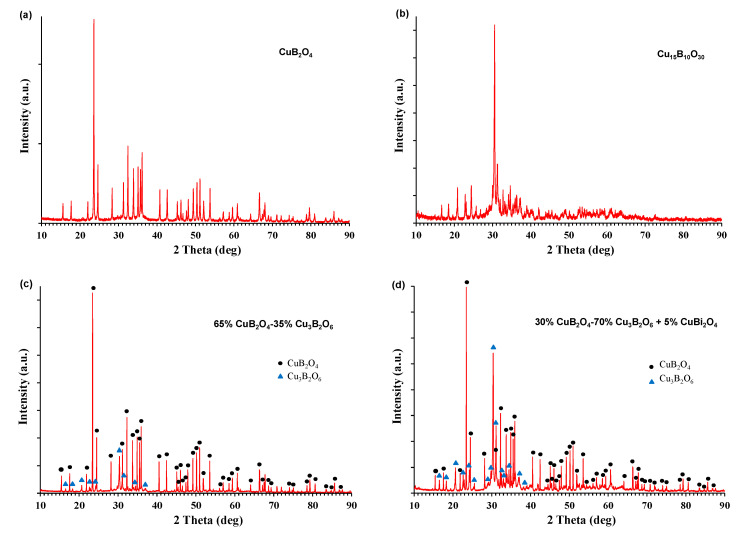
Diffraction patterns of the ceramics: (**a**) CuB_2_O_4_, (**b**) Cu_3_B_2_O_6_, (c) 65% CuB_2_O_4_–35% Cu_3_B_2_O_6_, and (**d**) 30% CuB_2_O_4_–70% Cu_3_B_2_O_6_ doped with 5% CuBi_2_O_4_.

As illustrated in Figure 1a,b, the XRD phase analysis confirmed the presence of the planned copper borates CuB_2_O_4_ and Cu_3_B_2_O_6_ as crystalline phases. CuB_2_O_4_ shows the tetragonal structure with the space group I-42, while Cu_3_B_2_O_6_ was detected as triclinic Cu_15_B_10_O_30_ with the space group P-1. For the compositions prepared as CuB_2_O_4_–Cu_3_B_2_O_6_ mixtures with a 5% CuBi_2_O_4_ addition, the XRD analysis revealed two main crystalline copper borate phases, but additional crystalline phases were not detected (Figure 1d). This implies that the sintering aid, CuBi_2_O_4_, formed an amorphous phase or entered the crystal lattice of the main crystalline components.

### 3.2. Heating Microscope Studies

**Figure 2:**
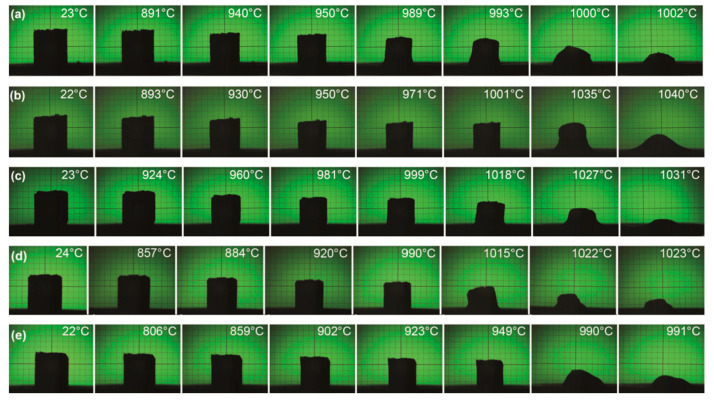
Selected images from a heating microscope for: (**a**) CuB_2_O_4_, (**b**) Cu_3_B_2_O_6_, (**c**) 65% CuB_2_O_4_–35% Cu_3_B_2_O_6_, (**d**) 50% CuB_2_O_4_–50% Cu_3_B_2_O_6_ doped with 5% CuBi_2_O_4_, (**e**) 30% CuB_2_O_4_–70% Cu_3_B_2_O_6_ doped with 5% CuBi_2_O_4_.

Figure 2 presents some selected images from a heating microscope that provided insight into the behavior of the samples during heating from room temperature to 1040 °C. 

These studies helped to establish the optimal firing profiles for each composition based on information about the temperature range in which the shrinkage occurs and about the softening and melting points. For pure copper borates (Figure 2a,b), the samples start to shrink at 891 and 893 °C, and the relevant optimal sintering temperatures are 940 and 930 °C for CuB_2_O_4_ and Cu_3_B_2_O_6_, respectively. The melting points are 1000 °C for CuB_2_O_4_ and 1040 °C for Cu_3_B_2_O_6_. The Cu_3_B_2_O_6_ ceramic shows a higher melting point than CuB_2_O_4_, but it has a similar temperature of the shrinkage onset and exhibits an advantageous feature of a broader sintering range. Consequently, its optimal sintering temperature is close or even lower as compared with CuB_2_O_4_. For mixed copper borates, the optimal sintering temperatures were established as 960, 920, and 900 °C for 65% CuB_2_O_4_–35% Cu_3_B_2_O_6_, 50% CuB_2_O_4_–50% Cu_3_B_2_O_6_ with 5% CuBi_2_O_4_, and 30% CuB_2_O_4_–70% Cu_3_B_2_O_6_ with 5% CuBi_2_O_4_, respectively.

### 3.3. Microstructural Studies

**Figure 3:**
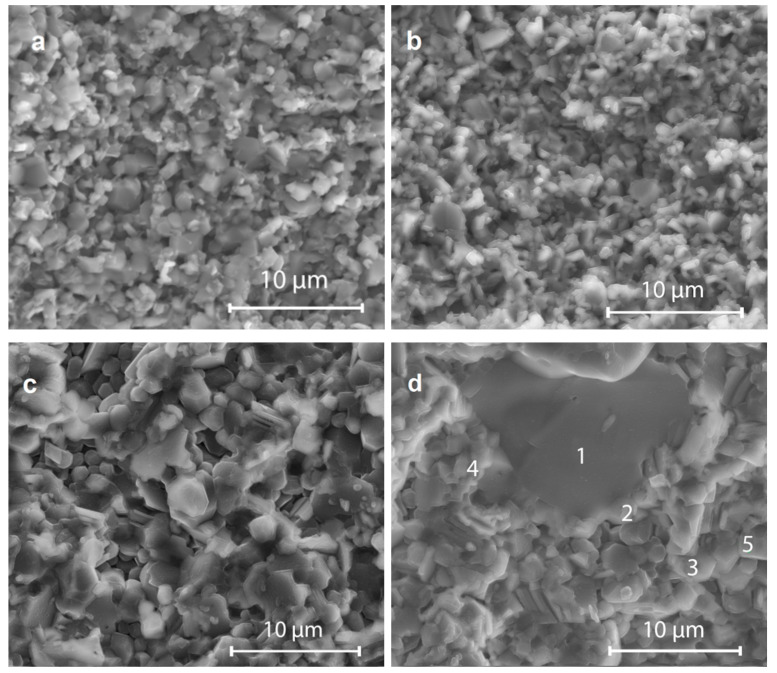
SEM images of fractured cross-sections of ceramic samples: (**a**) CuB_2_O_4_ sintered at 940 °C, (**b**) Cu_3_B_2_O_6_ sintered at 930 °C, (**c**) 50% CuB_2_O_4_–50% Cu_3_B_2_O_6_ doped with 5% CuBi_2_O_4_ sintered at 920 °C, and (**d**) 30% CuB_2_O_4_–70% Cu_3_B_2_O_6_ doped with 5% CuBi_2_O_4_ sintered at 900 °C, ×10,000.

The SEM studies of all the sintered samples based on pure and mixed copper borates showed a very compact microstructure with a small contribution of porosity. It follows from the comparison of the images in Figure 3a,b that the microstructure for pure copper borates is similar, fine-grained, and uniform, with grain sizes in the 0.5–3 μm range. 

For the mixed borates compositions, the dense microstructure was preserved, although there was a more significant variation in grain sizes as compared with the single-phase copper borate ceramics. For the ceramics with 5% CuBi_2_O_4_ added (Figure 3c,d), small grains 1–3 μm in diameter prevail, although a fraction of much bigger grains appears with sizes ranging from 4 to 12 μm. Thus, it seems that the sintering aid causes a grain growth effect, even though the sintering temperature is slightly lower as compared with pure copper borates. Table 1 presents the results of the EDS analysis at the points marked in Figure 3d for 30% CuB_2_O_4_–70% Cu_3_B_2_O_6_ ceramic doped with 5% CuBi_2_O_4_. Point one represents a big grain attributed to CuB_2_O_4_ (Cu/B ratio close to 0.5), while points two, four, and five were assigned to smaller grains of Cu_3_B_2_O_6_ (Cu/B ratio close to 1.5). Grain boundaries were enriched with Bi originating from the dopant CuBi_2_O_4_ (point three). The EDS results are distorted due to the imprecise detection of boron using this method.

### 3.4. Dielectric Properties

A theoretical prediction of dielectric permittivity based on the knowledge about the composition and crystal structure of the compound should be considered to design a substrate material with dielectric properties tailored for high frequency applications. For a simple assessment of the real part of relative dielectric permittivity *ε_r_*, one can use the Clausius–Mossotti equation, which relates this quantity with the polarizability *α*:

α =4πV_m_/3[(ε_r_ − 1)( ε_r_ + 2)] (1)

where *V_m_* is the molar volume.

For a compound, molecular polarizability can be calculated using the additive rule, as a sum of the polarizabilities of particular ions that built the molecule. Thus, the molecular polarizabilities of the investigated copper borates can be expressed as follows:

α(CuB_2_O_4_) = α(Cu^2+^) + 2α(B^3+^) +4α(O^2−^) (2)

α(Cu_3_B_2_O_6_) = 3α(Cu^2+^) + 2α(B^3+^) +6α(O^2−^) (3)

The polarizabilities of the constituent ions are 2.11, 0.05, and 2.01 Å^3^ for Cu^2+^, B^3+^, and O^2−^, respectively [40]. The molar volumes (calculated as the unit cell volume per the number of formula units in the unit cell) are 61.76 and 112.54 Å^3^ for CuB_2_O_4_ and triclinic Cu_3_B_2_O_6_, respectively. Thus, the theoretical relative dielectric permittivities of CuB_2_O_4_ and Cu_3_B_2_O_6_ calculated from the Clausius–Mossotti equation are 7.83 and 7.61, respectively. These values are close to each other.

However, the predictions based on the Clausius–Mossotti relationship are consistent with the experimentally measured values mainly for a high symmetry cubic crystallographic system. For the materials characterized by structural peculiarities related to the presence of “rattling” or “compressed” cations, ionic or electronic conductivity, dipolar impurities, or piezoelectric behavior, distinct deviations from the additivity rule were observed [40].

Low polarizability is responsible for confining ionic polarization in a material. A lower average bond length diminishes the rattling effect of cations in a polyhedral structural unit. A lower cell volume restricts the interaction of polarizable dipoles [41,42,43]. Qin et al. [41] proposed a universal model based on machine learning for predicting microwave dielectric permittivity. These authors stated that there are three most important features related to the crystal structure of a compound determining its dielectric permittivity. According to this model, the dielectric permittivity decreases with a decrease in the polarizability per unit cell volume *ppv* and with a decrease in the average bond length *blm.* The average cell volume per atom *va* is also an important parameter that should be maintained in an optimal range. Qin et al. [41] stated that the ranges of the decisive parameters that favor creating materials with a low dielectric permittivity are *ppm* < 0.15, *va* 11–16 Å^3^, and *blm* < 2.3 Å.

The relevant values for CuB_2_O_4_ and Cu_3_B_2_O_6_ obtained in this work are 0.17 and 0.16 for *ppm*, and 17.6 and 10.2 for *va*, respectively. The bond lengths reported for CuB_2_O_4_ are 1.999 Å for prevailing Cu–O shorter bonds, 2.864 Å for Cu–O longer bonds, and 1.444–1.487 Å for B–O bonds [26]. For Cu_3_B_2_O_6_, the average Cu–O bond length is 2.1 Å [28]_._ The analysis of *ppm*, *va*, and *blm* values for CuB_2_O_4_ and Cu_3_B_2_O_6_ leads to the conclusion that these parameters are close to the ranges indicated in [41] for low permittivity candidate materials.

**Figure 4:**
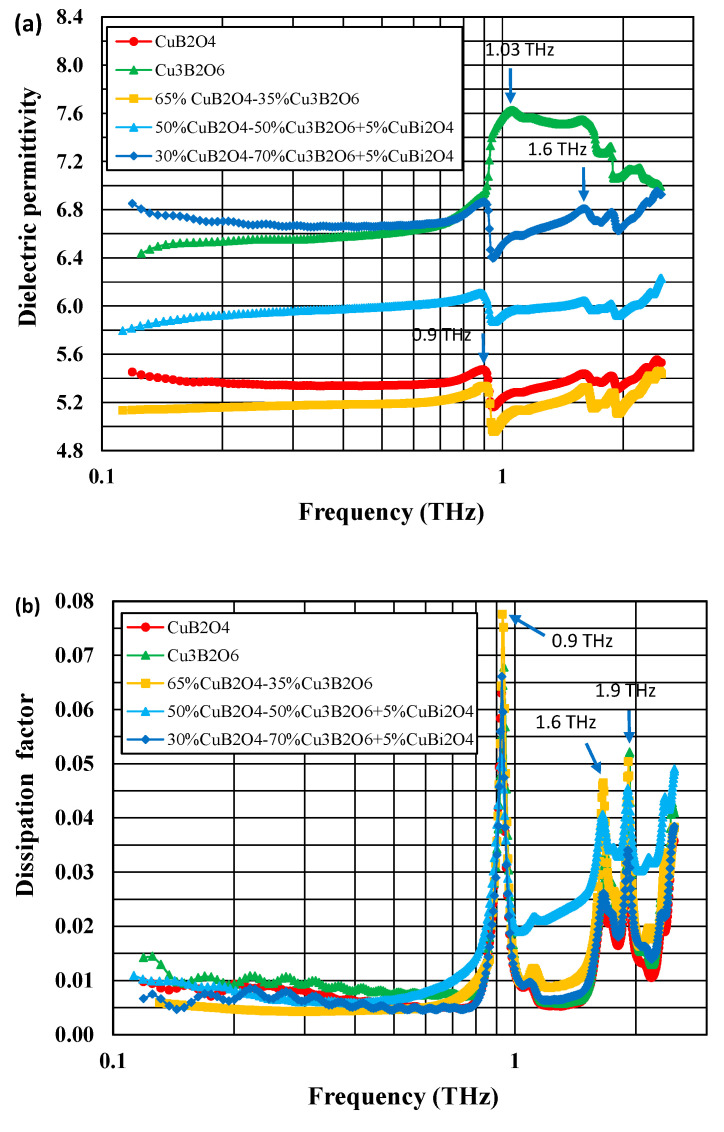
Comparison of dielectric permittivity (**a**) and dissipation factor (**b**) at 20 °C as a function of frequency in the range 0.12–2.5 THz for CuB_2_O_4_, Cu_3_B_2_O_6_, and CuB_2_O_4_–Cu_3_B_2_O_6_ ceramics.

Figure 4a,b compare the frequency dependences of the dielectric permittivities and the dissipation factors of copper borate ceramics at 20 °C in the 0.12–2.5 THz range. 

In the 0.14–0.7 THz range, the dielectric permittivities are low, at a level of 5.3–5.4 for CuB_2_O_4_, 6.4–6.7 for Cu_3_B_2_O_6_, 5.1–5.2 for 65% CuB_2_O_4–_35% Cu_3_B_2_O_6_, 5.8–6.0 for 50% CuB_2_O_4–_50% Cu_3_B_2_O_6_ with 5% CuBi_2_O_4_, and 5.8–6.1 for 30% CuB_2_O_4–_70% Cu_3_B_2_O_6_ with 5% CuBi_2_O_4_. The lowest dielectric permittivities were shown by pure CuB_2_O_4_ ceramic and 65% CuB_2_O_4–_35% Cu_3_B_2_O_6_ ceramic without the sintering aid. For all the materials under investigation, the dielectric permittivity changes very slightly with a frequency up to 0.7 THz and then reaches a maximum at about 1 THz for Cu_3_B_2_O_6_ and at about 0.9 THz for the rest of the copper borate-based ceramics.

**Figure 5:**
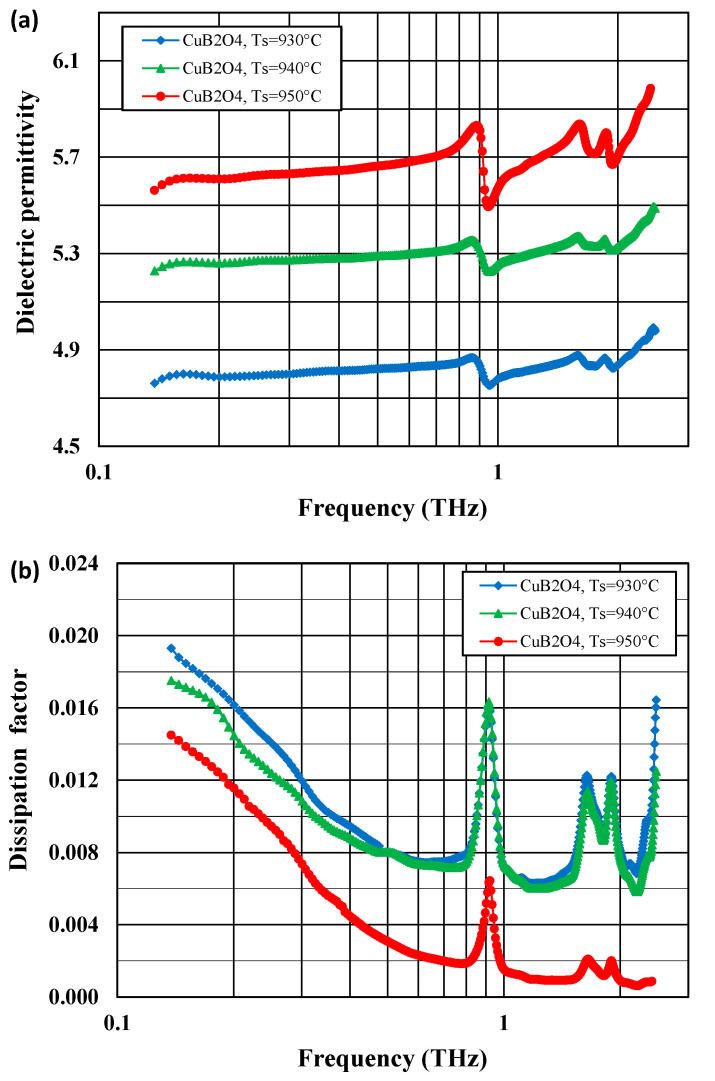
Comparison of dielectric permittivity (**a**) and dissipation factor (**b**) versus frequency in the range 0.12–2.5 THz for CuB_2_O_4_ ceramics sintered at 930, 940, and 950 °C.

Figure 5a,b show the comparison of the dielectric permittivities and dissipation factors of the CuB_2_O_4_ ceramics sintered at three different temperatures—930, 940, and 950 °C. The dielectric permittivity increases, while the dissipation factor decreases with an increasing sintering temperature. This is typical behavior that can be attributed to a lower porosity of the samples sintered at higher temperatures.

The dissipation factors are relatively low (0.004–0.01) in the 0.14–0.7 THz range, with a flat minimum at 0.4–0.6 THz. A few peaks on the dissipation factor versus frequency plots were observed above 0.9 THz at the positions corresponding to those of the dielectric permittivity maxima.

At very high THz frequencies, some types of dielectric polarization, such as space charge and dipolar polarizations, cannot follow the changes of the external electrical field. In this case, the dielectric behavior is determined by ionic, atomic, and electronic polarization. The dielectric properties can be described by the damped harmonic oscillators model [44]. This model explains the observed frequency independent constant value of the real part of dielectric permittivity ε’, an increase in its imaginary part *ε”* and, consequently, the dissipation factor (*ε”/ε’*) in the region of THz frequencies.

Peaks on the dielectric permittivity/dissipation factor versus frequency plots that occur above 0.7 THz are supposed to be attributed to phonon modes related to vibrations in Cu–O complexes [26,28]. Due to the large number of atoms that form the unit cells of both copper borates (42 atoms for CuB_2_O_4_, 110 atoms for Cu_3_B_2_O_6_ [26,28]), phonon modes for these compounds are numerous, which was confirmed using infrared and Raman spectroscopic studies [26,27,28,29].

**Figure 6:**
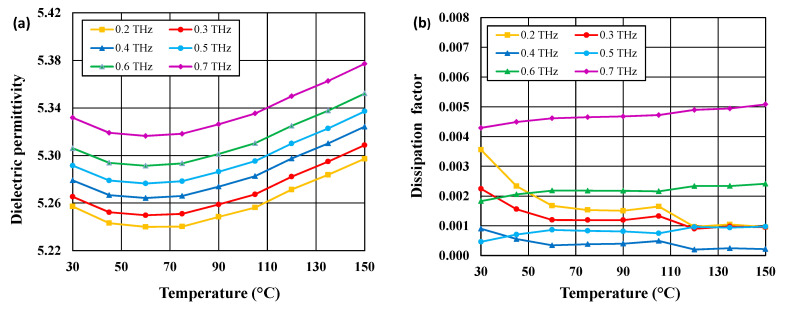
Comparison of dielectric permittivity (**a**) and dissipation factor (**b**) of CuB_2_O_4_ ceramic sintered at 940 °C as a function of temperature in the range 30–150 °C for a few frequencies in the 0.2–0.7 THz range.

In Figure 6a,b, the dielectric permittivities and dissipation factors for a few frequencies in the 0.2–0.7 Hz range (the region of a weak frequency dependence) are plotted as a function of temperature in the range 30–150 °C for the CuB_2_O_4_ ceramic. The temperature dependence of dielectric permittivity is very weak up to 90 °C, while the dissipation factor is almost temperature independent in the whole analyzed range. The frequencies corresponding to the peaks of dielectric permittivity and dissipation factor do not change with temperature, which implies that the phenomena responsible for these peaks are not thermally activated processes. It was found that the temperature coefficient of dielectric permittivity of CuB_2_O_4_ ceramic in the temperature range 30–90 °C is negative and changes from −19 to −55 ppm/°C in the 0.2–0.7 THz range.

The dielectric permittivities determined experimentally in this work are distinctly lower than those calculated using the Clausius–Mossotti equation. This discrepancy cannot be assigned only to porosity, considering the high relative density of the sintered samples at a level of 95–98%. It is supposed to be related to the complex noncentrosymmetric crystallographic structures of the copper borates under investigation. For such systems, deviations from the Clausius–Mossotti relationship have often been observed [40].

For commercially available LTCC materials, the values of dielectric permittivity in the range 4–7 and tan*δ* below 0.012 at 1 THz are considered low values, suitable for millimeter wave systems. The dielectric properties of CuB_2_O_4_ and CuB_2_O_4_–Cu_3_B_2_O_6_ ceramics in the 0.14–0.7 THz range are comparable with those reported for the commercial LTCC material Ferro A6M at 1 THz *(ε_r_’* = 6.06, tan*δ* = 0.012) [45]. We plan to use the developed powders based on copper borates for tape casting and the fabrication of multilayer LTCC substrates appropriate for very high frequency applications in future work. 

## 4. Conclusions

New ceramics based on two copper borates, CuB_2_O_4_ and Cu_3_B_2_O_6_, were successfully prepared via solid state synthesis and sintering processes. These ceramics exhibit the following advantageous features: a low sintering temperature suitable for LTCC technology, a very dense microstructure, a low and temperature stable dielectric permittivity (5.1–6.7), and a low dielectric loss (0.004–0.01) in the 0.14–0.7 THz range. The developed ceramics are promising substrate materials for submillimeter wave applications and have been investigated for the first time in such a frequency range. 

## Figures and Tables

**Table 1:** Results of EDS analysis at the points marked in Figure 3d for 30% CuB_2_O_4_–70% Cu_3_B_2_O_6_ ceramic doped with 5% CuBi_2_O_4_, sintered at 900 °C.

Element	at. %				
	Point 1	Point 2	Point 3	Point 4	Point 5
B	45.76	26.06	28.20	31.63	26.31
O	31.40	16.75	25.15	26.65	17.11
Bi	0.20	0.62	0.92	0.47	0.74
Cu	22.64	56.57	45.73	41.25	55.84
Cu/B	0.49	2.17	1.62	1.30	2.12

## Data Availability

The data presented in this study are available on request from the corresponding author. The data are not publicly available as the data also form part of an ongoing study.
